# A Knowledge-Based Algorithm for Automatic Monitoring of Orthodontic Treatment: The Dental Monitoring System. Two Cases

**DOI:** 10.3390/s21051856

**Published:** 2021-03-07

**Authors:** Silvia Caruso, Sara Caruso, Marianna Pellegrino, Rayan Skafi, Alessandro Nota, Simona Tecco

**Affiliations:** 1 Department of Life, Health and Environmental Sciences, University of L’Aquila, 67100 L’Aquila, Italy; silvia.caruso@univaq.it (S.C.); saracaruso2704@gmail.com (S.C.); 2 Private Practice in Dentistry, 81100 Caserta, Italy; mariannapellegrino95@gmail.com; 3 Private Practice in Dentistry, 75001 Paris, France; rraysk@gmail.com; 4 Dental School, Vita-Salute San Raffaele University and IRCCS San Raffaele Hospital, 20132 Milano, Italy; nota.alessandro@hsr.it

**Keywords:** artificial intelligence, app, orthodontics, smartphone, teledentistry, interceptive orthodontics, removable orthodontic appliance

## Abstract

Background: In the dental field, digital technology has created new opportunities for orthodontists to integrate their clinical practice, and for patients to collect information about orthodontics and their treatment, which is called “teledentistry.” Dental monitoring (DM) is a recently introduced orthodontic application that combines safe teledentistry with artificial intelligence (AI) using a knowledge-based algorithm, allowing an accurate semi-automatic monitoring of the treatment. Dental Monitoring is the world’s first SaaS (Software as a Service) application designed for remote monitoring of dental treatment, developed in Paris, France, with Philippe Salah as the Co-founder and CEO. Cases presentation: This report describes two cases in which DM system was essential to achieve the control of certain movements: it was possible to follow the movement, even if complex, such as the anterior cross of an adult patient and a lack of space in the canine of the growing patient. The software analyzed the fit and retention of the aligner, thus ensuring correct biomechanics. They were treated during the COVID-19 pandemic lockdown with aligners. The first case is a growing patient who was monitored during an interceptive orthodontic treatment to manage a retained upper canine. The second case is an adult patient forced to finalize his treatment of upper lateral incisor crossbite. The software analyzed the fit and retention of the aligner, thus ensuring correct biomechanics. Conclusions: DM system appears to be a promising method, useful for improving the interaction between doctor and patient, generally acceptable and useful to patients, even in critical clinical situations, at least in cases with optimal compliance and ability to use the tool properly.

## 1. Introduction

Continuous evolution of digital technology changes everyday life, both on a professional and personal level. There is unlimited access to information; on-demand services have become the new norm, and remote work is no longer a challenge. One of these innovative ways is through the smartphone, which makes interaction between patient and doctor more accessible and convenient. According to the literature, smartphone acceptance and the need of medical apps to enhance educational and services are high [1,2]. These software apps create new opportunities for doctors to integrate technology into their clinical practice, and for patients to collect information about their treatment [3,4,5]. In this way, the patient is constantly involved in their treatment journey, not only during the treatment choice. All these opportunities are called “telemedicine” or “eHealth”and “teledentistry” in the dental field. The concept of “teledentistry” is not a distant reality anymore: according to recent surveys, 78% of patients will start using teledentistry within the next five years [6]. By definition, teledentistry is the implementation of electronic information, imaging and communication technologies, including interactive audio, video and data communications, as stated by the American Teledentistry Association (https://www.americanteledentistry.org/ (accessed on 10 January 2021)). It provides a support to dental care delivery, diagnosis, consultation, treatment and patient education. It can include virtual consultations and remote monitoring of patients, offering less expensive and more convenient care options for both patients and doctors. A number of systematic reviews examining the outcomes and costs associated with teledentistry have been published [2]. Petya et al. revealed that 89% of patients strongly agree that interactive dental care can improve their oral health, and this can be easily obtained with some dental apps [7]. For orthodontic discipline, teledentistry offers a fundamental strategy to engage the patient in order to drive compliance. The possibility of having a two-way communication channel with the patient allows to provide feedback, give positive reinforcement and keep them informed of the progress of the treatment. This constitutes an improvement in terms of compliance and treatment success.

Recently, an innovation has been introduced in orthodontics, called Dental Monitoring (Dental Mind, Paris, France). Dental monitoring (DM) is a recently introduced orthodontic application that combines safe teledentistry with artificial intelligence (AI) using a knowledge-based algorithm, allowing an accurate semi-automatic monitoring of the treatment. This is the world’s first SaaS (software as a Service) application designed for remote monitoring of dental treatment. It was developed in Paris, France, and Philippe Salah is the Co-founder and CEO. The company has commercial (not academic) interests, with 31 patents on the various mechanisms of the system, and a first personal patent on remote monitoring. In fact, this DM system is available all over the world, in all European languages as well as in Chinese and Japanese.

It is an AI-powered system, respecting the Health Insurance Portability and Accountability Act (HIPAA) and the General Data Protection Regulation (GDPR), that allows the orthodontist to remotely monitor the patient’s treatment through intra-oral pictures taken by the patient with their smartphone and a special ScanBox© (a patented device that allows the patient to take high quality orthodontic pictures).

The DM system was created to satisfy the need to monitor patients remotely, but it can be adapted to all patients, who can then be followed with greater precision during the treatment. For example, the hygiene of our patients or the status of the orthodontic treatment can be evaluated remotely.

In the DM, an AI powered system runs a preliminary assessment of data from intra-oral pictures taken by the patients using a knowledge-based algorithm. Thus, it partially automates communication among doctor, staff, and patient. Differently from other dental apps [8], it gives real-time validated information thanks to the knowledge-based algorithm. In general, technology positively impacts rapid response, error prevention (on 110 hand transcribed lists and 90 electronically generated medication lists reviewed for errors by a pharmacist, the findings showed significantly fewer errors with the electronically generated discharge lists, 8%, compared with the hand transcribed lists, 22%; *p* < 0.05), information accessibility, and data management (documentation via the handheld device recorded significantly more diagnoses per patient-median diagnoses = 9—compared with paper documentation—median = 4; *p* < 0.0001), as assessed by a recent systematic review [9].

It is known that during the COVID-19 lockdown, thousands of orthodontic patients in epidemic areas missed their monthly visits because of the suspension of all non-urgent procedures. This treatment interruption and the potentially related problems caused concerns for both patients and orthodontists [10].

According to Sharif et al., only 7% of patients are aware that apps are available to help with their orthodontic treatment, and 87% of them would like to use an app to support their orthodontic therapy [11]. Those data highlight how the current awareness of the availability of apps is poor and that the patient desire to have them is significant. This should make clinicians consider the great potential of such an effective app.

Recently, there has been an increase in the availability of orthodontic apps. The majority of them are patient-focused and are most commonly for entertainment rather than clinical purposes. Only a small number of apps aim to elicit a behavioral change in patients. Therefore, there is the need to assess the quality and educational content of them [12]. In response to the need to improve the interaction between doctor and patient, the DM system seems to be a promising solution. An early review suggests that this technology leads to improved clinical outcomes, highest patient satisfaction, as well as cost savings [13].

Differently from other telecommunications systems such as Skype, Google Duo, Zoom, etc. [14], which cannot provide a standardized evaluation of clinical situation, the DM system provides process automation through a knowledge-based algorithm that is based on a combination of robotic and deep learning process, with information systems that act like a semi-intelligent user.

The present report describes two cases where this technology has brought added benefits to the traditional orthodontic treatment. The patients were an adult patient and a growing patient, both undergoing orthodontic treatment with aligners. The adult patient managed to follow the treatment at a distance despite the complexity of the movements; the growing patient was monitored during the loss of deciduous teeth, and also managed to follow the treatment.

## 2. The Dental Monitoring System

**Figure 1:**
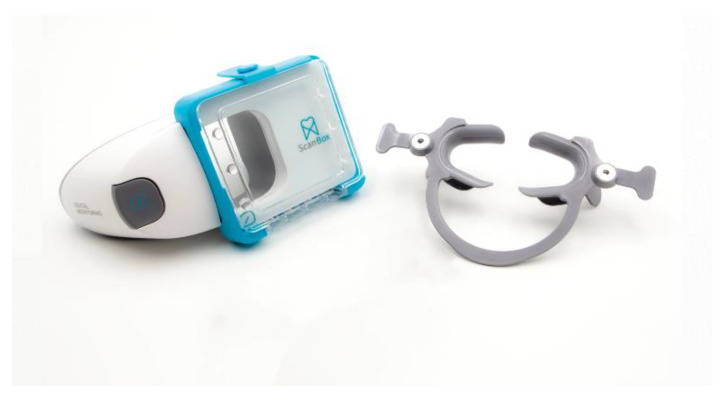
Dental monitoring (DM) ScanBox© and cheek retractor.

A complete DM system is based on the following steps. First, the DM system requests that the patient is provided with a cheek retractor and a DM ScanBox© (Figure 1). These are designed to facilitate and standardize the process of taking intra-oral pictures by the patient with their smartphone, to ensure an acceptable quality of clinical intra-oral images. Instructions are given, including short tutorial video for each step, in order to guide the patient. A whole scanning process takes just a few minutes, and every kind of smartphone is compatible with the DM system and the patient’s age. Then, the patient is asked to download the DM app, to follow the protocol chosen by the orthodontist.

**Figure 2:**
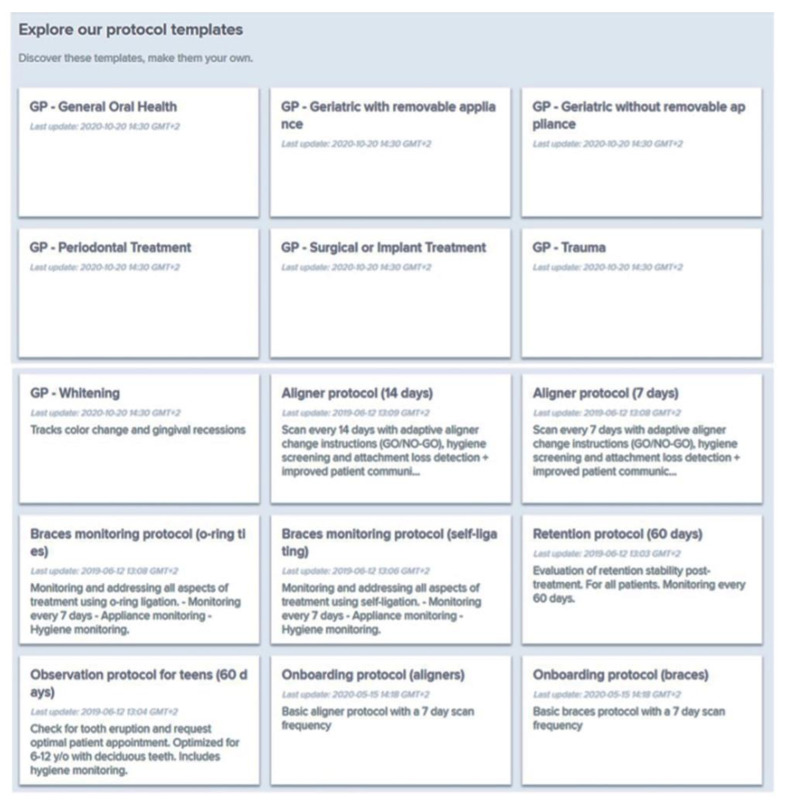
Protocols template in the DM system. The DM system can be used to monitor different kinds of treatments (not only orthodontics): prosthetic dentistry, surgery, traumatic injury, periodontal treatments, dental bleaching, oral hygiene, and general oral health.

**Figure 3:**
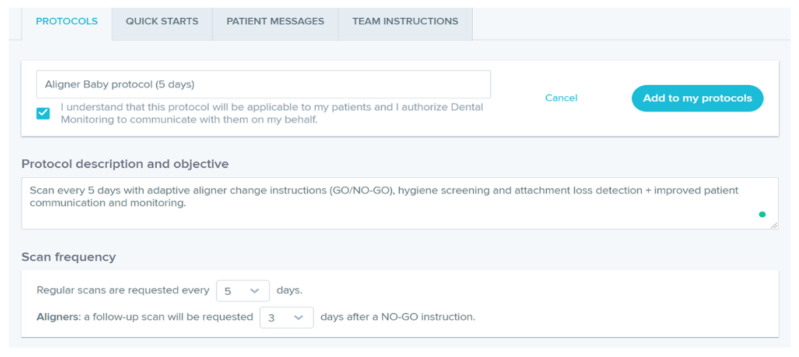
Personalized protocol used by the orthodontist.

**Figure 4:**
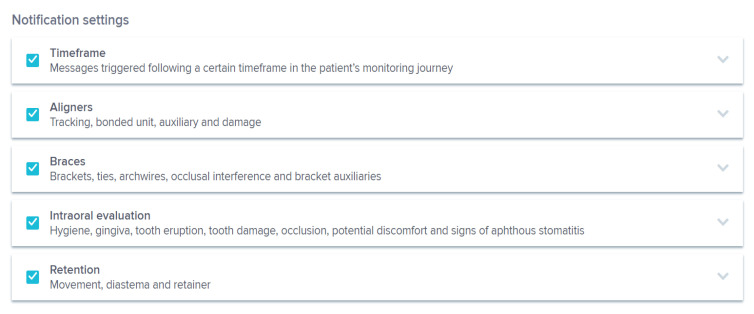
Notification settings in a DM system protocol.

The orthodontist can choose a protocol among those proposed by the DM system (Figure 2), or create new customized protocols for each patient (Figure 3). A protocol is basically a set of commands that inform the AI powered system on what to monitor and notify to clinicians (Figure 4) and how to process the information, with everything being fully customizable and editable at any moment.

The DM has four different protocols based on the different characteristics and specificities of each protocol, from the simplest to the most complex. They are: Photo Monitoring Light (limited to two scans per month, NO clinical analysis, only self-assessment required, and message to/from patients); Photo Monitoring (2D clinical analyses, unlimited scans, dynamic replacement of aligners, “Go Live”); 3D Monitoring Light (3D calculations of dental movements and 2D clinical analyses, limited to one scan per month, incompatible with the dynamic replacement of aligners); 3D Monitoring Full (3D calculations of dental movements, 2D clinical analysis, and unlimited scans, incompatible with the dynamic replacement of aligners). There are different prices, according to the protocol: Photo Monitoring Light, 6 euro/month, Photo Monitoring, 9.90 euro/month, 3D Monitoring Light, 12.90 euro/month, 3D Monitoring Full, 15.90 euro/month. The orthodontist can also create new customized protocols for each patient.

In orthodontics, DM system protocols can include the automatic detection of the debonding of braces, tubes, bands, or buttons, damage to hooks or temporary anchorage device ligatures, archwire disengagement, occlusal interferences with an archwire or a bracket, oral hygiene, soft tissues inflammation, gingival recessions, tooth damage, signs of aphthous stomatitis, dental eruption, appliance cleaning, black triangles, and stability of fixed and removable retainers.

**Figure 5:**
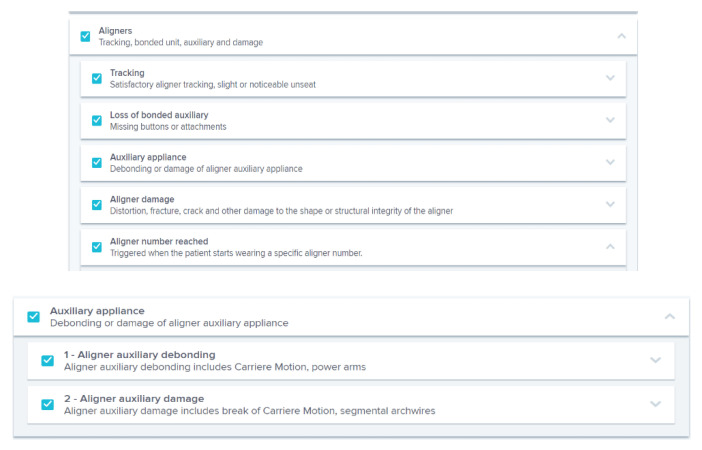

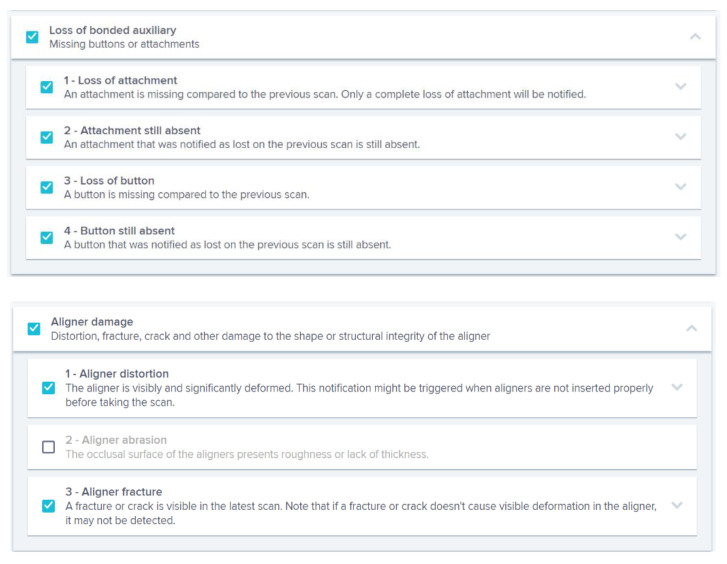
Protocol for treatment of the aligners by the DM system.

In particular, during an orthodontic treatment with clear aligners, remote monitoring should be carried out at each aligner change, in order to allow the clinician to evaluate the fit and ensure perfect adaptability of the aligner, the presence or integrity of attachments, the presence and maintenance of buttons and elastics, and the integrity of teeth and aligners (Figure 5). The follow-up information is given to the patient by the DM system, on the basis of a preliminary analysis made by the AI thanks to a knowledge-based algorithm. For the treatment with clear aligners, immediately after the intraoral scan taken by the patient, the DM app tells the patient if they can change the aligner and start to use the next one (signal “GO”) or if they should keep the current one (signal “No Go”). In case of a “No Go” signal, a new intraoral scan can be required after a few days. The knowledge-based algorithm is based on a combination of robotic process automation and deep learning process. The robotic process automation relies on an integration among workflow, rules and ‘presentation layer,’ with information systems that act like a semi-intelligent user of the systems [15]. The deep learning process is the most complex and refined form of machine learning and artificial intelligence and it is based on a neural network [16]. It includes a training set early by bio engineering, and the DM assessment is based on it. Applying deep learning process in dentistry has taken a long time from dedicated AI engineering to optimize and collect sufficient data (millions of dental images) to feed the machines in early stages, label the images one by one, and eventually build the neural networks that make the machine semi “intelligent”. Labeling included images about various clinical situations, concerning: debonding of braces, tubes, bands, or buttons, damage to hooks or temporary anchorage device ligatures, archwire disengagement, occlusal interferences with an archwire or a bracket, oral hygiene, soft tissues inflammation, gingival recessions, tooth damage, signs of aphthous stomatitis, dental eruption, appliance cleaning, black triangles, fixed and removable retainers stability, the fit and perfect adaptability of the aligner, the presence or integrity of attachments, the presence and maintenance of buttons and elastics, and the integrity of teeth and aligners. The AI looks for these clinical situations.

The transmission of the information from the patient’s mouth to the servers happens remotely. When the patient is prompted to scan, they will receive a notification, according to the chosen protocol, and then the guided scanning process will send between 20 and 30 images to the servers for processing, which can be summarized in four different steps:

Step 1: The raw images are processed by the system. They are screened for quality in order to determine if the patient needs to take another scan or not.
Step 2: The system is able to detect teeth and identify them with a prediction score (percentage of certainty). The technology is so advanced that in some cases it is able to differentiate if the tooth is a first or second premolar in cases of orthodontic extraction. The gingiva is also detected.
Step 3: Detection of the different clinical parameters.
Step 4: The AI will analyze the data and will then send instructions to the patient and the team based on the chosen protocol.

Then, the orthodontist is notified when a situation that requires a clinical appointment appears. The interaction among patient/app/dentist is continuous, because the clinician can always monitor the images sent by patients and there is a notification when clinical attention is needed.

## 3. Cases Presentation

### 3.1. Case 1

**Figure 6:**
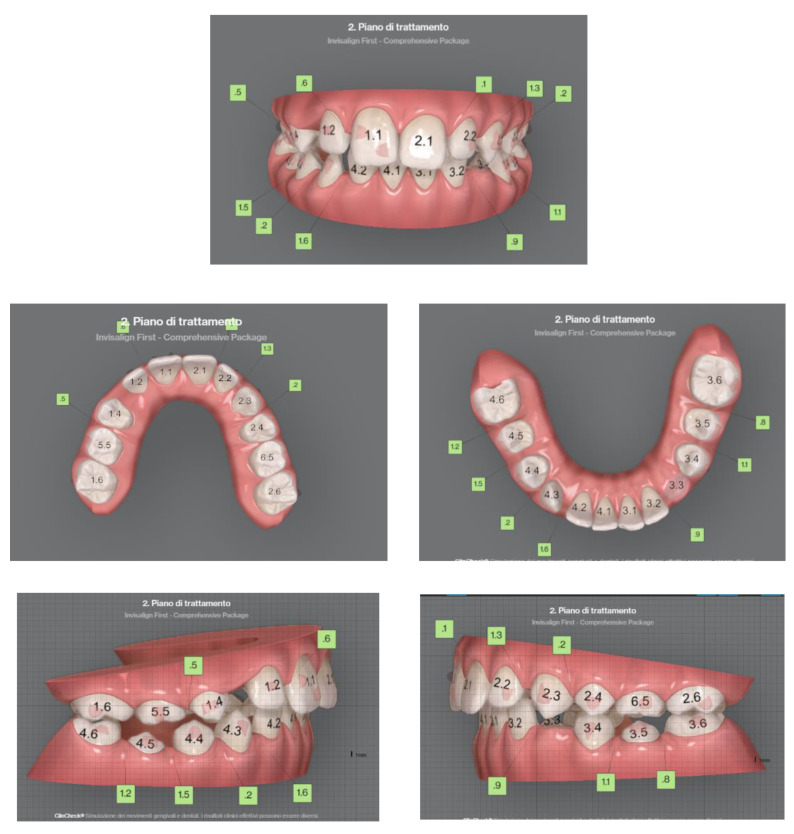
Clinical case treated with Invisalign© aligners. Intraoral views from ClinCheck. Frontal, upper, and lower occlusal and lateral view of occlusion before the beginning of treatment.

**Figure 7:**
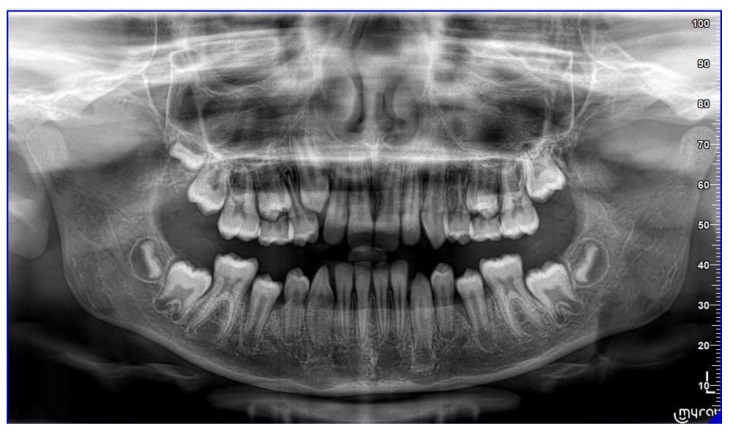
Pre-treatment Orthopantomography.

The patient is an 11-year-old female, in late mixed dentition. Clinical examination revealed acceptable oral hygiene, absence of functional anomalies and oral habits, the presence of deciduous molars (5.5 and 6.5), and erupting permanent teeth. The intraoral examination showed a molar and canine Angle class II on both sides, increased overjet, posterior unilateral functional crossbite on the right side, lower/upper midline deviated to the right/left of 2 mm, and increased overbite (5 mm) (Figure 6). The most important problem was that 1.3 was not in the arch, while the contralateral 2.3 had been present for more than 6 months. Diagnostic X-ray confirmed the 1.3 eruption delay. (Figure 7).

The 1.3 delayed eruption was probably due to the 1.4 mesial inclination and the absence of space. The patient, as demonstrated by cephalometric analysis and radiographic examination, was very near the growth peak, indicating the need to begin the treatment to obtain success of the orthodontic therapy.

#### 3.1.1. Treatment

The orthodontic therapy included a dentoalveolar expansion of the upper arch, to obtain a spontaneous eruption of 1.3 in dental arch, avoiding surgical disocclusion. Thus, an immediate treatment with clear aligners was planned.

Other aims of the treatment were: molar and canine Angle class correction, correct overjet, overbite, and crossbite resolution, with a harmonious alignment of all teeth.

A Clincheck with Invisalign was approved, which included 32 aligners. In the present case a customized “Aligner Baby protocol (5 days)” was selected, that means the patient had to change her clear aligners every 5 days.

The growth spurt of the patient indicated the necessity to immediately begin the therapeutic protocol. Unfortunately, the start of treatment for this patient coincided with the 2020 lockdown for the COVID-19 pandemic in Italy (February–June 2020). Consequently, just as the arch expansion and the 1.3 eruption were beginning, the patient would have had to stop the treatment. This could have entailed a complication, because if the canine 1.3 had erupted in an arch that was still very narrow and without space, the patient would subsequently have to face a mechanic of “distalization” instead of a simple alignment or interceptive expansion, or worse, a surgical disinclusion.

In the present case, thanks to the DM system, it was possible to monitor the treatment remotely, and constantly receive photographic reports, crucial for a clinical evaluation at a time when a remote evaluation was not only useful for a regular orthodontic check-up, but extremely essential with respect to therapeutic timing and properly manage the eruption of the upper right canine 1.3. A further advantage of DM is its ease of use. The growing patient at first required the help of her mother to perform the scans, then continued alone thanks to the practicality of the software.

#### 3.1.2. Results

**Figure 8:**
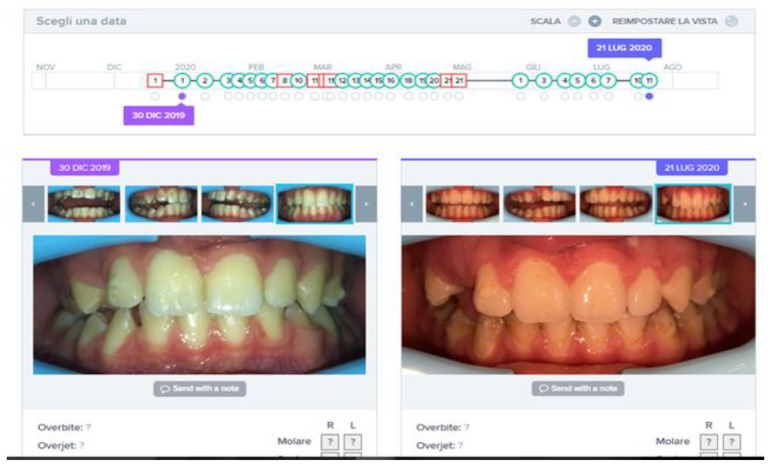
Sequence of monitoring over time for Case 1, with Go signals in green and No Go signals in red. At the end of the period, after 7 months of treatment, the space in the arch for tooth 1.3 is perfectly obtained.

The patient reached a good teeth alignment in less than 7 months during the pandemic lockdown in Italy. The molar and canine (only on the left side) class I was reached, the midline discrepancy was significantly improved, and the posterior crossbite on the right side was corrected with a dentoalveolar expansion of the upper arch. The space on the arch for the 1.3 element was fully created. During her treatment, the patient received almost exclusively “GO” signals (Figure 8), which means that she showed good compliance with clear aligners and confidence with the DM system.

### 3.2. Case 2

**Figure 9:**
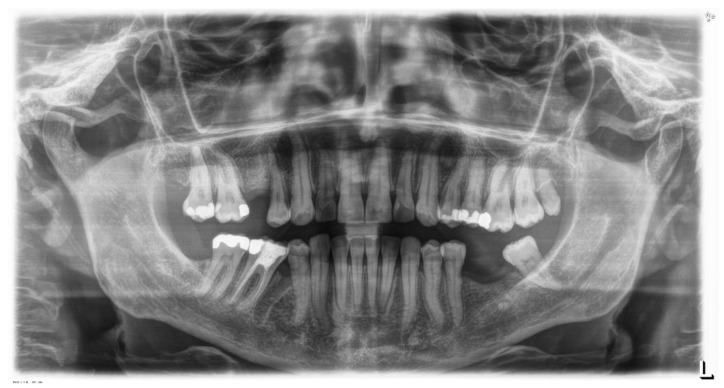
Pre-treatment orthopantomography.

**Figure 10:**
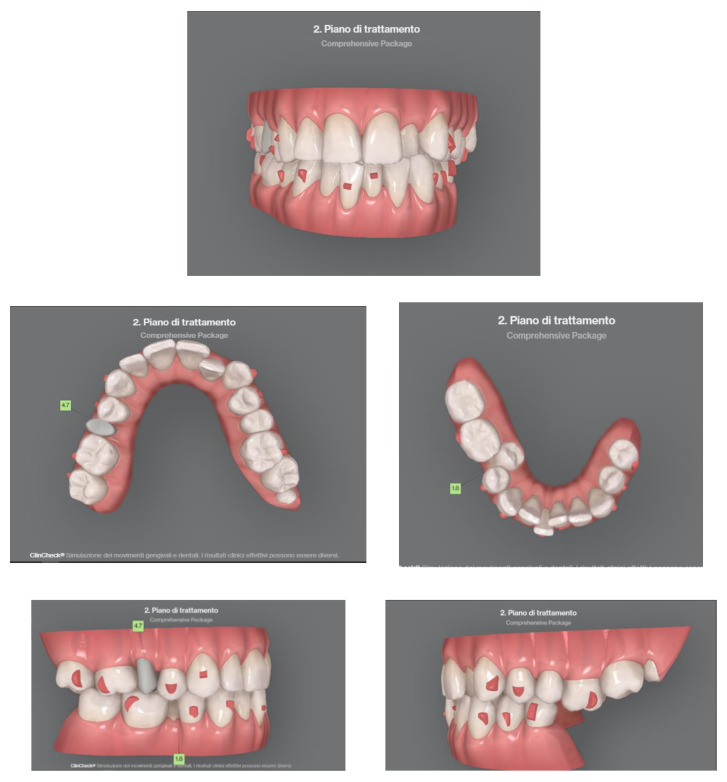
Clinical case treated with Invisalign^©^ aligners. Intraoral views from ClinCheck. Frontal, upper, and lower occlusal and lateral view of occlusion before the beginning of treatment.

Case 2 concerns a 57-year-old male, with no systemic diseases. He came to our observation in November 2019, to carry out an orthodontic treatment to improve the esthetic appearance of his smile. He showed a good oral hygiene and periodontal condition. Intra-oral evaluation showed a reduction of the transverse diameters, both in the upper and lower dental arches, and a lack of space for the tooth 4.5 in the lower arch (Figure 9 and Figure 10). It was planned to re-establish elements 1.6 and 3.6 after orthodontic therapy in order to reach the first molar class. The most important problem for the patient was related to his smile esthetics and concerned the crossbite of the tooth 2.2.

His specific request was to obtain a noticeable improvement in his smile as soon as possible, with the resolution of the anterior crossbite. It was decided to use aligners to obtain esthetic and functional goals.

#### 3.2.1. Treatment

In the first phase, the patient had to wear 23 aligners with a 7-day aligner protocol. The choice to change aligners every 7 days in an adult patient is strongly related to compliance, and requests excellent collaboration by the patient in following the orthodontist prescriptions, without which the clinician would have to significantly extend the time of treatment. In this case, the DM system allowed to strictly follow patient compliance and monitor the resolution of the anterior cross that could be an insidious problem if not carefully monitored. In the present case, the No Go notification, in fact, was often linked to the complexity of the teeth movements, which could not be monitored without the DM, as some aligners required more time to obtain the desired result. The DM system allowed to obtain the proper planned results and allowed to avoid interruptions.

#### 3.2.2. Results

**Figure 11:**
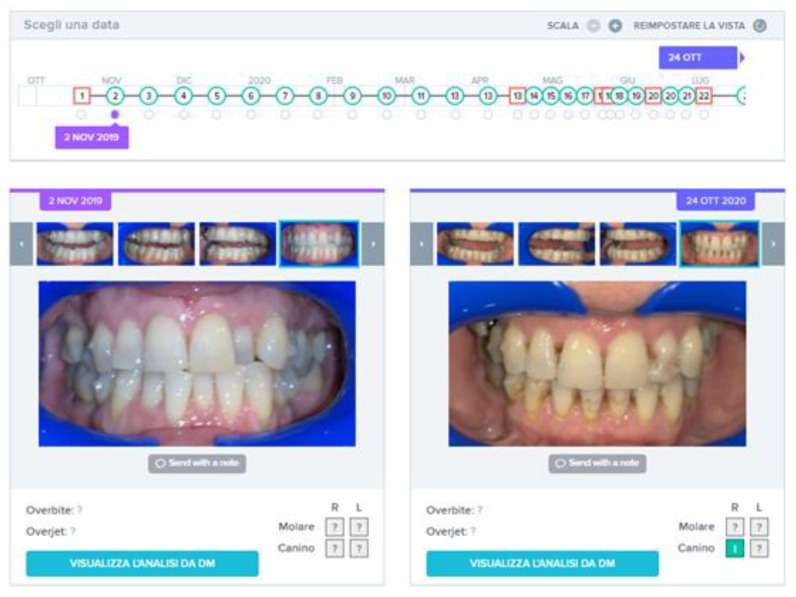
Sequence of monitoring over time for Case 1, with Go signals in green and No Go signals in red. At the end of the period, after 12 months of treatment, the tooth 2.2 is perfectly aligned.

The patient reached good teeth alignment in 12 months. The crossbite on 2.2 was treated, reaching an ideal alignment. Molar and canine class I was reached, the midline was centered and the crowding was significantly improved. During his treatment, the patient received almost all “GO” signals (Figure 11), which means that he showed an excellent compliance and confidence with the DM system. Monitoring was made active until all clinical goals were achieved. It was, therefore, suspended only while waiting for the new aligners.

## 4. Discussion

The DM system allows to monitor orthodontic treatment and improves its efficiency. It improves the patient’s motivation and compliance, it optimizes time and allows to intervene promptly in case of unexpected events, such as debonding or damage of the orthodontic device, through a constant monitoring of the therapy. The DM system allows the patients to see their mouth and therapeutic progresses whenever they want. Furthermore, a continuous interaction between the patient and the orthodontist is possible; the orthodontist can ask the patient for an immediate scan, send them a message, or give them clinical instructions. This gives the patient a sense of constant “care” and maximum availability from the orthodontist and makes them feel they are a participant in their orthodontic treatment. In the present case of an adult patient, the communication allowed to immediately obtain explanations about the necessity to continue to wear the same aligner, which happened when the patient received the No Go communication. Other communications allowed to immediately clarify procedures about hygiene, aligner cleaning, and pain during treatment.

According to the present clinical results, it can be said that the DM system was effectively capable of improving the experience of the patients who were undergoing orthodontic treatment, obtaining, in these situations, results that probably could not have been achieved with the traditional method.

In the present clinical cases, the DM system was crucial and essential to achieve an appropriate interceptive therapy in a growing subject, thus avoiding a more complex treatment (in case 1), and to closely monitor (every 7 days) an adult patient who was in a hurry to improve the esthetics of his smile (in case 2). It can be concluded for these particular cases that without the DM system, it would not have been possible to achieve the same results, considering the period of the pandemic lockdown, which increased the use of virtual consultation [17]. Above all, the orthodontic treatments were monitored with periodic scans at every alignment change.

Regarding patient satisfaction, most of the treated patients find the use of dental monitoring advantageous. In particular, the patients of the two cases presented reported their considerations: the adult patient informed us that he found the app very easy to use. Although it was new to him, he found everything very clear. The message he received according to our protocols were precise and timely and the analysis of the AI allowed him to change the aligners without interfering with the treatment. According to the growing patient, doing the scans was very easy; initially, she took needed help from her mother, later she managed to do it alone. The mother also reported that monitoring the presence or absence of the elements to be included in the orthodontic treatment was important for managing appointments.

Potential scenarios for this methodology include the treatment of patients who live very far from the dentist, the possibility for any patient to be clinically followed by those they consider to be the best specialists in the world, with an easy access to excellent centers of healthcare, and the management of cases during lockdown.

Possible weaknesses of the method, which need to be verified over time, are: the accuracy of monitoring on a 3D photo as opposed to clinical probing—including the use of touch or instruments—of the oral cavity and the limit of the method adopted for taking intra-oral photos that could be, at least for some patients, not optimal, despite the tutorial. Therefore, a learning curve by patients may be required.

## 5. Conclusions

The DM system appears to be a promising method, useful for improving the interaction between doctor and patient, significantly increasing treatment efficiency, even in critical clinical situations, at least in cases with optimal compliance and ability to use the tool properly.

## Data Availability

The data presented in this study are available on request from the corresponding author. The data are not publicly available due to privacy.
